# Associations of HLA DR and DQ molecules with Lyme borreliosis in Latvian patients

**DOI:** 10.1186/1756-0500-5-438

**Published:** 2012-08-14

**Authors:** Lilija Kovalchuka, Jelena Eglite, Irina Lucenko, Mara Zalite, Ludmila Viksna, Angelika Krumiņa

**Affiliations:** 1 Riga Stradiņš University, Clinical Immunology and Immunogenetic laboratory, Kronvalda str.9, Riga, Latvia; 2 Riga Stradiņš University, Infectology and Dermatology department, Linezera 3, Riga, Latvia; 3 Riga Stradiņš University Infectology and Dermatology Department, Infectology Center of Latvia, Linezera 3, Riga, Latvia

**Keywords:** Lyme borreliosis, HLA alleles, PCR

## Abstract

**Background:**

Many autoimmune diseases are associated with variants of HLA genes such as those encoding the MHC complex. This correlation is not absolute, but may help in understanding of the molecular mechanism of disease. The purpose of this study was to determine HLA-DR,-DQ alleles in Latvian patients with Lyme borreliosis and control (healthy) persons. Case patients and control subjects were similar in age, gender and ethnic heritage and differed only as regards the presence of *Borrelia burgdorferi* infection. The study included 25 patients with clinical stage *– erythema migrans* and 30 control (healthy) persons. HLA genotyping was performed by PCR with sequence-specific primers.

**Results:**

The results show difference in HLA-DRB1 alleles distribution between patients and control subjects. The frequencies of HLA-DRB1 *04 (OR 11.24; p < 0.007) and HLA-DRB1 *17 (03) (OR 8.05; p < 0.033) were increased in the Lyme disease patients. And the frequency of allele DRB1*13 (OR 0.12; p < 0.017) was lower in Borreliosis patients and higher in control group. But, significant differences in frequencies of HLA-DQ alleles we did not detect.

**Conclusions:**

HLA predisposition to Lyme borreliosis appears not to be limited to HLA molecules, but some HLA-DR alleles also have a significant influence, and, may have implications in our understanding of pathogenesis of this disease. In particular, HLA-DRB1*04 and DRB1 *17 (03) may contribute to the Lyme borreliosis development in Latvian population

## Background

Lyme disease is a tick-borne multisystem disease that affects primarily the skin, nervous system, heart and joints. At least three species of *Borrelia burgdorferi* sensu lato, namely *Borrelia burgdorferi* sensu stricto, *Borrelia garinii*, and *Borrelia afzelii*, can cause the disease.

The illness may evolve in stages, beginning with *erythema migrans* and progressing through a stage of dissemination during which arthritic, neurological, and cardiac complications may occur. Some patients with Lyme borreliosis experience recurrent episodes of joint inflammation for months or years. Although the pathogenesis of this condition is unclear, several lines of evidence suggest autoimmunity. The histology of synovial lesions in Lyme borreliosis is similar to that for rheumatoid arthritis (RA) and includes hyperplasia, vascular proliferation, and lymphoid infiltrates [1]. The majority of individuals with Lyme disease have the HLA-DRB1*0401 or HLA-DRB1*0101 allele, alleles which also occur more frequently in patients with RA [2]. Furthermore, while *Borrelia burgdorferi* DNA can be detected in joint fluid of Lyme disease patients by PCR prior to treatment with antibiotics, it is unusual to detect such DNA in synovium or synovial fluid after antibiotic treatment, especially for patients experiencing recurrent Lyme arthritis [2,3]. These findings suggest that the pathogenesis of joint disease in chronic Lyme borreliosis may be a result of antibody directed against a component of the *Borrelia burgdorferi* spirochete that cross-reacts with synovial tissue.

Lyme disease is the most common vector-borne disease in the United States [3,4], the incidence remains high in Latvia also. During last 10 years (2001–2010), tick-borne encephalitis morbidity in Latvia varies from 6.2 to 22.3 per 100.000 inhabitants (142 to 494 people per year). Lyme borreliosis morbidity in Latvia during the same time period was between 14 and 36.9 per 100.000 inhabitants (328 to 829 people per year). The largest number of cases of Lyme borreliosis was recorded in 2010 year - 829 [5]. Latvia is considered to be an endemic territory; however, people get affected in other countries of the world as well. The maximum incidence rates in patients were observed in the age group 60–69 [6,7].

In Europe most cases occur in Scandinavia and Central Europe. A prospective, population-based survey in southern Sweden revealed an annual incidence of 69 cases per 100,000 populations [8]. As in the United States, there were areas of endemicity in which the annual incidence reached 160 per 100.000 inhabitants [8]. A similar study in southern Germany found an annual incidence of 111 cases per 100,000 inhabitants.

The purpose of this study was to determine HLA-DR,-DQ molecules in patients with clinical, epidemiological and laboratory approved Lyme borreliosis diagnosis.

## Materials and methods

The study included 25 patients (8 males, 17 females; aged between 35 and 74 years) with clinical stage – *erythema migrans* and 30 control (healthy) persons (12 males, 18 females; aged between 21 and 57 years). All patients and healthy persons are residents of Latvia. The clinical diagnosis was confirmed at Infectology Center of Latvia. Immunogenetic examinations were performed in Riga Stradiņš University, Clinical Immunology and Immunogenetic laboratory. The Riga Stradinš University Ethics Committee approval was obtained (September 9, 2010). And the writteninformed consent for participation in the study from participants was obtained.

Genomic DNA was extracted from proteinase-K-treated peripheral blood leukocytes using the routine “salting-out” method [9,10]. The DNA was stored in TE buffer (10 ml Tris–HCl, pH 7.5, and 2 ml 0.5 M Na2 EDTA per liter of distilled water). The DNA concentration, around 100–200 μg/ml, was determined by fluorescence with a DNA fluorimeter.

### HLA- typing

HLA-DR genotyping by PCR Low-resolution for DRB1*01 to DRB1*18; HLA-DQA1 typing for DQA1*0101 to DQA1*0601; and HLA-DQB1 typing for DQB1*0201 to DQB1*0608 was performed by PCR with sequence-specific primers (PCR–SSP) [10,11]. The reaction mixture (15 μl) included 1.0 μl DNA, 1.5 μl PCR buffer [50 mM KCl, 1.5 mM MgCl2, 10 mM Tris–HCl (pH 8.3)], 0.6 μl dNTPs (25 mmol/l), 1.0 μl specific primers (0.2 mmol/l), and 0.5 U of the *Taq* DNA polymerase (Promega). The reaction mixture was subjected to 35 amplification cycles, each consisting of one denaturation cycle at 94°C (60 s), seven annealing cycles at 94°C (40 s) and 67°C (15 s), and final 28 extension cycles at 93°C (10 s) and 65°C (9 s). PCR products were visualized by agarose-gel electrophoresis [10,11]. After addition of 2 M loading buffer, the PCR reaction mixtures were loaded in agarose gels prestained with ethidium bromide (0.5 μk/ml gel). Gels were run for 15 min at 10 V/cm gel in 0.5 mM TBE (0.89 M Tris, 0.89 M Boric acid and 0.02 M EDTA in aqueous solution) buffer and then examined under UV illumination and recorded [11].

### Statistical analysis

The significance of differences in individual subtypes between patients and controls was assessed by Fisher exact test for small numbers [12] and with Bonferroni correction. Odds ratios (OR) and 95% confidence intervals (CI) were computed by standard methods [12].

## Results

Typing of all sixteen alleles DRB1were investigated (Table1). The frequency of HLA-DRB1 *04 (OR 11.24; p < 0.007) and HLA-DRB1 *17(03) (OR 8.05; p < 0.033) were increased in the Lyme disease patients compared with the control groups. The frequency of allele DRB1*13 (OR 0.12; p < 0.017) was lower in Borreliosis patients and higher in controls. After Bonferroni adjustment the difference was no significant when pc-value was 0.106, 0.415, and 0.239 (respectively) (Table1).

**Table 1:** The frequency of DRB1* alleles studied in-patients and healthy controls from Latvia

**Allele DRB1**	**Patients (n = 25)**	**Controls (n = 30)**	**OR**	***p-value***	***pc-value***
			**(95% CI)**		
	**50 alleles**	**60 alleles**			
*01	0	5 (17%)	ND	ND	
*02	0	2 (7%)	ND	ND	
*03	3 (12%)	4 (13%)	0.89 (0.21-3.83)	0.601	
*04	8 (32%)	1 (3%)	**11.24** (1.24-74.18)	0.007	0.106
*07	3 (12%)	5 (17%)	0.70 (0.18-2.87)	0.464	
*08	2 (8%)	3 (10%)	0.79 (0.14-4.60)	0.586	
*09	2 (8%)	3 (10%)	0.79 (0.14-4.60)	0.586	
*10	1 (4%)	3 (10%)	0.39 (0.04-3.73)	0.380	
*11	6 (24%)	9 (30%)	0.77 (0.31-2.09)	0.648	
*12	3 (12%)	4 (13%)	0.89 (0.21-3.83)	0.601	
*13	1 (4%)	9 (30%)	**0.12** (0.02-1.02)	0.017	0.239
*14	0	2 (7%)	ND	ND	
*15	4 (16%)	1 (3%)	5.13 (0.55-41.58)	0.130	
*16	2 (8%)	2 (7%)	1.21 (0.18-8.22)	0.619	
*17(03)	6 (24%)	1 (3%)	**8.05** (0.90-57.83)	0.033	0.415
*18(03)	3 (12%)	1 (3%)	3.77 (0.39-33.54)	0.243	

*Abbreviations:* ND - not defined; OR (odds ratio); CI (confidence interval); p-value (probability); pc-value (after Bonferroni adjustment).

Further we focused on the HLA DQA1 and HLA-DQB1 genotyping. HLA-DQA1 typing for DQA1* 0101, *0102, *0103, *0201, *0301, *0501, *0401/*0601; and HLA-DQB1 typing for DQB1* 0201, *0301, *0302, *0303, *0304, *0305, *0501, *0601, *0602/*0608 alleles were studied also. We did not detect significant differences in frequencies of HLA-DQ alleles. Although, the HLA-DQA1*0501(OR 3.77; p < 0.243), and DQB1*0201(OR 3.22; p < 0.151) were increased in patients compared with the control groups, but the difference was no significant (data not shown).

## Discussion

Lyme disease is a multi-faceted illness, initiated upon infection with the spirochete *Borrelia burgdorferi*. One manifestation of the disease is arthritis, which can result in chronic arthritis in a small subset of exposed individuals [13]. The prevalence of HLA-DR4 related alleles in these patients is an indication of an autoimmune process [13,14].

Many autoimmune diseases are linked to variants of HLA genes such as those encoding the MHC class II complex [15]. Chronic Lyme arthritis is associated with MHC class II variants that are able to bind to fragments of the *Borrelia burgdorferi* protein OspA (outer surface protein A) encompassing amino acid residues 165 through 173. Antigen-presenting cells whose MHC class II molecules display OspA165-173 peptides on their surface stimulate T cells that recognize the OspA peptide [16,17].

The first hypothesis that Lyme arthritis may have an autoimmune component is the demonstration of its association with the HLA–DR4 and HLA–DR2 alleles [14,18]. Patients who have HLA–DRB molecules that bind an epitope of *Borrelia burgdorferi* outer surface protein A (OspA163–175) are more likely to have chronic arthritis than are patients with other DRB molecules [19,20]. Molecular techniques have identified the OspA163–175–binding molecules as the rheumatoid arthritis (RA) alleles (DRB1*0401, *0404, *0405, *0101, *0102) [21,22] and the DRB5*0101 allele linked to DRB1*1501 (the former DR2 allele) [22]. Moreover, patients with chronic arthritis often have T cell recognition of OspA163–175 [23,24]. In other words, simply the presence of the DR4 allele was enough to trigger an inflammatory immune response.

Why is this particular gene so important? The DR allele is responsible for presenting the antigen to the T cells for an immune system response. When the antigen is presented to the T cell in the context of the DR4 allele, T cells are stimulated to produce interferon gamma, an inflammatory response. In contrast, when the antigen is presented in the context of the DR11 allele, it stimulates the production of antibodies, a response that does not induce inflammation. The antibodies bind to the bacteria and eliminate it. So somehow the presence of either the DR4 or DR11 allele determines the T cell response — whether to produce interferon gamma and launch an inflammatory attack or to produce antibodies to the antigen, instead [25]. In the case of DR4, once the immune response has begun, it is self-perpetuating because interferon gamma will stimulate T cells to produce more interferon gamma. Thus the initial immune response determines the outcome of the disease in the long run.

In our HLA study, an association was confirmed between Lyme borreliosis and the HLA-DRB1 *04 and -DRB1 *17(03) (part of the older HLA-DR3). The distribution of alleles in the patients included in this study follows the world tendency: HLA-DRB1 *04 and DRB1 *17(03) was the most frequent allele in Caucasian population. Thereafter, we began to determine HLA-DQ alleles in Latvian patients with Lyme arthritis. The HLA-DQA1*0501 and DQB1*0201 were increased in patients, but, significant differences in frequencies of HLA-DQ alleles we did not detect.

## Conclusions

These results suggest that the inflammatory events of the subacute arthritis can set the stage for development of chronic disease in individuals possessing an HLA susceptibility allele. In particular, HLA-DRB1*04 and DRB1 *17 (03) may contribute to the Lyme borreliosis development in Latvian population, and may have implications in our understanding of pathogenesis of this disease. To receive more reliable data on the prevalence of HLA alleles in Latvian population and their possible relationship with Borreliosis it is necessary to continue the investigation and extend the range of persons under investigation. Treatment for patients with acute manifestations of Lyme disease is well established and effective. Understanding the pathophysiology of chronic borreliosis and treatment-resistant Lyme arthritis are both major challenges and a prerequisite for the eventual development of effective treatments for these conditions.

This work was supported by Riga Stradiņš University grant 09.1604.

## Competing interest

The authors declare that they have no competing interests.

## Author’s contributions

LK carried out the HLA typing and drafted the manuscript. JE participated in the design of the study and performed the statistical analysis. IL described epidemiogical data and performed the statistical analysis. MZ enrolled patients in study and keep clinical and laboratory data base. LV conceived of the study and participated in its design and performed scientific literature resources. AK participated in the design of the study, coordination and helped to draft the manuscript. All authors read and approved the final manuscript.
